# Knowledge, Attitudes, and Practices Related to Artificial Intelligence Among Medical Students and Academics in Saudi Arabia: A Systematic Review

**DOI:** 10.7759/cureus.83437

**Published:** 2025-05-03

**Authors:** Zaki Alsahafi, Ahmaed Baashar

**Affiliations:** 1 Basic Sciences, College of Science and Health Professions, King Saud Bin Abdulaziz University for Health Sciences, Jeddah, SAU; 2 Research Office, King Abdullah International Medical Research Center, Jeddah, SAU

**Keywords:** artificial intelligence, educational technology, faculty training, medical education, saudi arabia

## Abstract

This systematic review aims to analyze the existing literature on artificial intelligence (AI) applications in medical education in Saudi Arabia, and it spanned the period from January 2020 to February 2025. The review focuses on the nature and scope of AI applications, evidence synthesis types, geographical distribution of authorship, quality of research, challenges encountered, and research gaps within Saudi Arabia. Studies were retrieved from the PubMed, Google Scholar, ProQuest, and Web of Science databases. The process followed the guidelines outlined by the Preferred Reporting Items for Systematic Reviews and Meta-Analyses (PRISMA). We included studies that explored knowledge, attitudes, and practices of AI among medical students and academics in Saudi Arabia. We first screened the titles and abstracts of the studies according to our inclusion criteria, and then reviewed the full texts of those that met the criteria. A standardized form was used to collect data, including author information, study population, research objectives, and key findings.

The review identified key areas of focus, including personalized learning, interactive simulations, and real-time feedback in medical education. Most studies discussed the potential benefits of AI tools in improving student engagement and clinical decision-making skills. However, significant challenges were reported, such as insufficiencies in faculty training, data privacy concerns, and disparities in technological infrastructure. While the use of AI in medical education in Saudi Arabia has great potential, there are still significant challenges. There is a need for proper training for faculty and standardized AI curricula. More research is required to assess the long-term effects of AI on educational outcomes and find ways to overcome the current barriers to its successful implementation.

## Introduction and background

The concept of artificial intelligence (AI) was initially described in 1956 by McCarthy, and Turing later expanded on the idea, defining AI as the presence of intelligent reasoning that could be integrated into machines [1,2,3]. Along with the advancements in AI proficiencies, the definition of AI has been continuously evolving [3]. With the advent of recent AI tools and technologies, including generative pre-trained transformers, natural language processing (NLP), expert systems, machine learning, intelligent agents, personalized learning, and virtual learning environments, AI has emerged to represent the capability of a digital machine to perform tasks that are typically associated with intelligent beings. These tasks include planning, diagnosing disease, summarizing, self-correcting, decision-making, creativity, and improving learning, teaching, assessment, and educational administration [3,4,5,6,7].

Integrating AI into the education system takes considerable effort and guidance. Its importance in education has been highlighted through various initiatives and reports at both the international and national levels. In the United States, for instance, organizations developing AI-based personalized learning platforms have received significant funding to improve student academic performance and reduce educational disparities for disadvantaged individuals [6,8,9]. Similarly, China has launched a strategic plan to modernize education by promoting the use of intelligent technology in classrooms and expanding professional development opportunities for educators in AI-related fields [6,10,11,12]. Building on these international developments, Saudi Arabia has also embraced AI integration, particularly through Vision 2030 and the National Transformation Program. These efforts are expected to enhance AI technology and innovation in education [3,13]. To make the most of these government efforts, the Organization for Economic Co-operation and Development (OECD) has recommended that educational researchers engage in applied research to advance educational practices [14].

Given AI’s potential in promoting education, the technology has become a key focus for educational researchers, policymakers, and health practitioners. However, when comparing the use of AI in education to other educational technologies like gamification and blended learning, research in AI tends to be fragmented and less structured. Therefore, further research is required to understand whether and how these emerging technologies and applications benefit education [6,15].

The unfamiliarity with AI technologies, along with the need for governance and regulations concerning privacy, ethical, legal issues, equity, security, and readiness, poses a challenge in effectively introducing and integrating AI tools into schools and universities [2,16-18]. Gordon et al. reviewed 278 publications covering various applications of AI in medical education, including admissions, teaching, assessment, and clinical reasoning, and found that most papers focused on the early adaptation phases of AI in education, with only a few reporting its role in driving long-term educational changes [19]. A scoping review provided a thematic analysis of 22 publications on the employment of AI in undergraduate medical education. The findings revealed a significant heterogeneity and poor consensus across studies, with no standardized framework for integrating AI into the undergraduate medical curriculum [20]. Preiksaitis and Rose (2023) reviewed 41 publications regarding generative AI in medical education and found a variety of applications, including self-directed teaching, simulation, and writing support. However, their review also highlighted significant concerns, such as academic integrity, the accuracy of information, and the potential negative impact of generative AI on learning outcomes [21].

Numerous systematic reviews have also analyzed papers on the use of AI in medical education, focusing on the trends in studies such as subject areas, geographical distribution, and textual patterns [22]. Others have investigated specific disciplines like languages, mathematics, and medicine [23], individual educational activities such as assessment [24], and particular applications including assistive robots, adaptive learning, or proctoring systems [25].

Literature suggests that the existing knowledge of AI tools in the educational system across the Gulf Cooperation Council countries, including Saudi Arabia, remains fragmented and incomplete [13,26], despite substantial investments and efforts to advance technology and innovation in the field [13]. It has been reported that AI assists the development of interactive simulations, virtual patients, and real-time feedback, which aid medical students in refining their clinical decision-making skills. Moreover, it increases accessibility to medical education, especially in remote areas, thus expanding opportunities for quality education nationwide.

Despite its benefits, the adoption of AI in medical education within Saudi Arabia encounters certain challenges, including inadequate faculty training in AI tools, concerns about data confidentiality, and the absence of structured AI education in medical curricula. Moreover, over-reliance on AI might reduce human interaction and critical thinking skills. Differences in technological infrastructure across institutions may also limit the widespread implementation of AI-based education [27-35]. However, these studies have focused on the cognitive aspects and perceptions of medical students regarding the use of AI in their teaching and learning. Therefore, a more comprehensive approach is required to fully examine the role of AI in the educational system in Saudi Arabia.

To our knowledge, the impact of AI tools on medical education within Saudi Arabia has not been systematically evaluated. Therefore, this systematic review aims to analyze the existing literature on AI applications in medical education in Saudi Arabia, during the period from January 2020 to February 2025. This review highlights the nature and scope of AI applications, types of evidence syntheses, geographical distribution of authorship, quality, challenges encountered, and existing research gaps. The research questions were developed through a step-by-step process. We began by searching the existing literature to identify key themes and gaps. Based on that, we drafted an initial list of possible questions. After some discussion and refinement, we settled on the final set to ensure they matched the aims of our review. These questions were chosen as they best reflected the main areas we wanted to explore regarding AI in medical education within Saudi Arabia.

Research questions

RQ1: What is the focus and extent of research on AI tools in medical education within Saudi Arabia?

RQ2: What types of evidence syntheses have been conducted in the context of AI integration into medical education in Saudi Arabia?

RQ3: What is the geographical distribution of AI-related research in medical education within Saudi Arabia?

RQ4: What is the quality of research on AI tools in medical education within Saudi Arabia?

RQ5: What are the concerns expressed by students and academics in Saudi Arabia regarding the implementation of AI tools in medical education?

RQ6: What are the current research gaps in the integration of AI within medical education?

## Review

Methods

**Figure 1:**
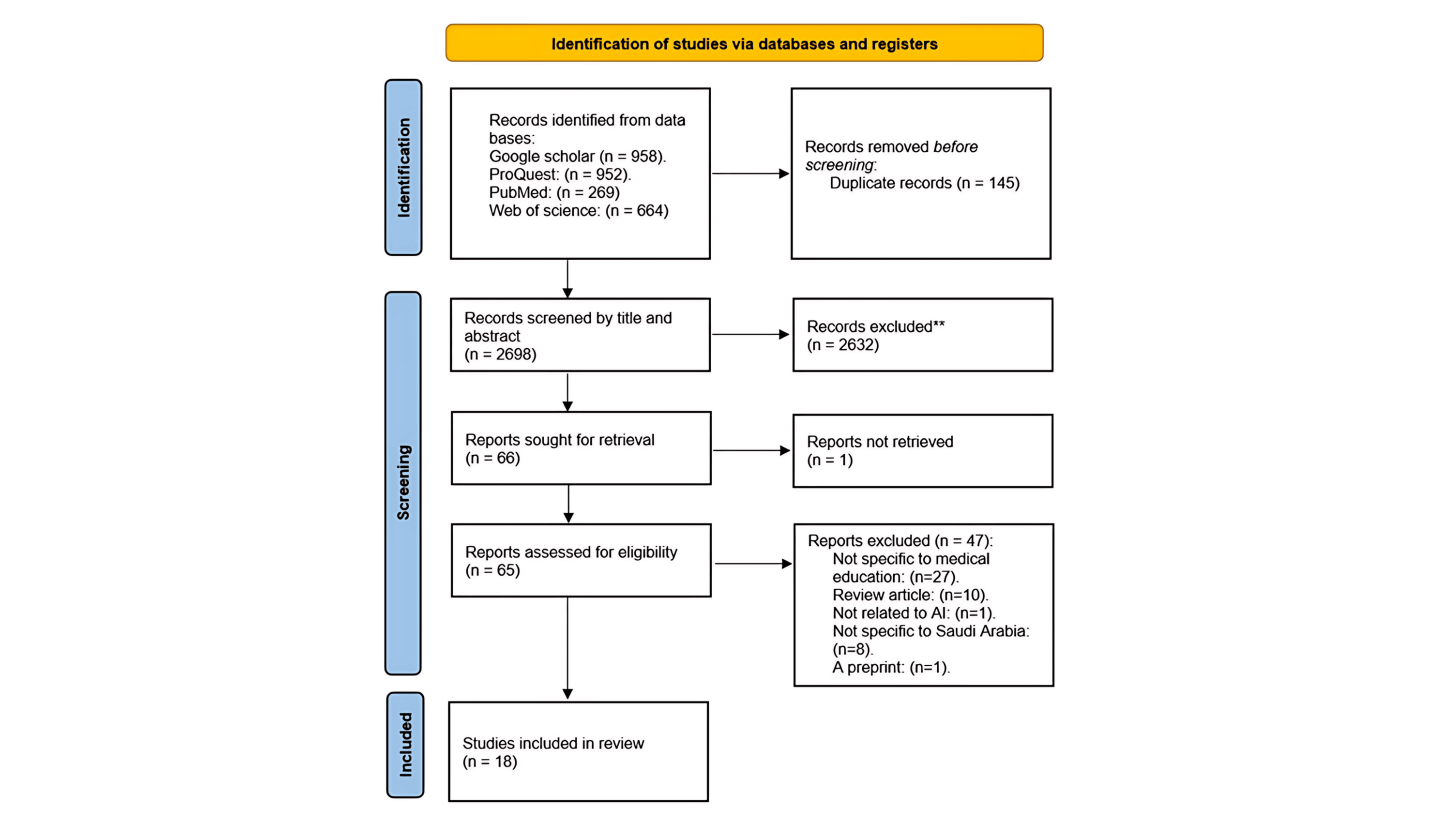
Flow diagram depicting the study selection process

This systematic review was based on the Preferred Reporting Items for Systematic Reviews and Meta-Analyses (PRISMA) 2020 guidelines, as illustrated in Figure 1 [36]. This study was approved by the King Abdullah Medical Research Center (KAIMRC) Institutional Review Board in Jeddah, Saudi Arabia (study number: NRJ25/068/3).

Study Design and Search Strategy

A comprehensive systematic review was conducted from February 2025 to March 2025, based on a reproducible search strategy. Primary research electronic databases, including PubMed, ProQuest, and Web of Science, were systematically searched. The detailed search strategy is outlined in Table 1. In addition, authors performed a search through Google Scholar using the terms “artificial intelligence” AND “medical education” AND “Saudi Arabia”.

**Table 1:** Review search string

Search string	
Artificial intelligence	“Artificial intelligence” OR “machine intelligence” OR “AI” OR “ChatGPT” OR “machine learning”
AND	
Medical education	"Medical education" OR "health professions education" OR "clinical education" OR "medical curriculum" OR “undergraduate medical students" OR "medical residents" OR "healthcare professionals" OR "medical faculty"
AND	
Saudi Arabia	"Saudi Arabia" OR "Kingdom of Saudi Arabia" OR "KSA" OR "Saudi"

Eligibility Criteria

This review analyzed only formally published primary research studies that investigated the attitudes, knowledge, and practices of medical students and academics regarding AI tools in medical education within Saudi Arabia. The screening process was conducted by the authors, who independently reviewed titles, abstracts, and full texts. The inclusion and exclusion criteria are detailed in Table 2. Only those research articles available as full text online were included. To ensure thorough coverage, we also reviewed the reference lists of the included papers and retrieved any additional articles that our search might have missed.

**Table 2:** Inclusion and exclusion criteria AI: artificial intelligence

Inclusion criteria	Exclusion criteria
Articles addressed the study objectives	Articles focusing on AI applications in diagnostics, professional training, or clinical practice
Articles written in the English language	Articles that include Saudi Arabia with other countries
Articles indexed in PubMed, ProQuest, or Web of Science	Articles tested an AI model for clinical purposes
Peer-reviewed original research articles	Articles where the population consists solely of healthcare professionals or clinicians
Articles focusing on AI applications in medical education within Saudi Arabia	
Articles published in the period between January 2020 and February 2025	

Data Extraction

Duplicate items were removed, and all documents gathered from the systematic search were compiled. The articles were then retrieved, and an initial screening of their titles and abstracts was conducted. Only those articles that met the inclusion criteria underwent a full-text review. The data extracted for this review include publication and authorship details (e.g., journal Q-rank, publication name, number of authors, and author affiliations), study objectives, characteristics of the study population and subjects, study design, and key findings.

Quality Assessment

To evaluate the quality of the studies analyzed, we used the Joanna Briggs Institute (JBI) critical appraisal checklist for cross-sectional and qualitative studies, as shown in Table 3. This tool assesses the methodological rigor of each study and helps identify potential biases in their design, execution, and analysis [37,38].

**Table 3:** JBI critical appraisal checklist items for cross-sectional and qualitative studies JBI: Joanna Briggs Institute

Cross-sectional studies	Qualitative studies
1 - Were the study objectives clearly stated? 2 - Were participants and settings appropriately selected? 3 - Was the sample size justified? 4 - Were exposure (independent variables) and outcome (dependent variables) measured reliably? 5 - Were confounding factors identified and appropriately controlled? 6 - Were valid and reliable measurement tools used? 7 - Was data analysis conducted with appropriate statistical methods? 8 - Were the results clearly presented?	1 - Is there congruity between the research methodology and the research question? 2 - Is the philosophical perspective stated and justified? 3 - Is the study design appropriate for the research question? 4 - Are the recruitment methods clearly described and appropriate? 5 - Is data collection clearly explained and justified? 6 - Is the data analysis process rigorous and aligned with qualitative methodologies? 7 - Are the findings supported by direct quotes or evidence from participants? 8 - Has the researcher considered their influence on the study? 9 - Are ethical considerations, including approval and informed consent, addressed? 10 - Do the conclusions align with the research findings?

We categorized the articles into two groups based on their methodology: cross-sectional studies or qualitative studies. Each study was then assessed using JBI’s 10-question checklist for cross-sectional studies or its eight-question checklist for qualitative studies. The responses were classified into four categories: yes, no, unclear, or not applicable. A "yes" response was given a score of 1, while "no," "unclear," and "not applicable" responses were scored as 0. Therefore, cross-sectional studies could achieve a maximum quality score of 10, while qualitative studies could achieve a maximum score of 8.

Data Synthesis

The analyzed studies employed a wide range of self-developed data collection tools, resulting in data that were too varied and inconsistent to be statistically combined for a quantitative analysis. As a result, performing a meta-analysis was not possible. Instead, we provide a thematic summary of the key findings across all studies. This allowed us to identify and highlight the main patterns and insights. Where applicable, the results are reported as mean ± standard error.

Results

Study Selection

As illustrated in Figure 1, 2,843 articles were identified across all databases. Initially, 145 duplicate records were removed. Then, following an initial screening of titles and abstracts, 2,632 articles were excluded. Subsequently, full-text articles were retrieved, with one being excluded due to unavailability. The remaining 67 articles underwent full-text screening. Based on this, 18 articles met the inclusion criteria and were included for full-text analysis.

*Quality Assessment* 

To assess the quality of the analyzed studies, the journal Quartile (Q) ranking of each article was first assessed (Table 4). Only three articles were published in Q1 journals [33,34,39], while most studies were published in Q3 or Q4 journals.

**Table 4:** Summary of Q-rank of the studies analyzed

Article	Journal name	Q-rank
Alharbi et al., 2024 [39]	Scientific Reports	Q1
Almarzouki et al., 2025 [33]	BMC Medical Education	Q1
Elhassan et al., 2025 [34]	JMIR Medical Education	Q1
Al Shahrani et al., 2024 [30]	Healthcare	Q2
Alwadaani et al., 2024 [31]	Journal of Multidisciplinary Healthcare	Q2
Bin Dahmash et al., 2020 [27]	The British Journal of Radiology	Q2
Syed and Alrawi, 2023 [40]	Medicina	Q2
Syed et al., 2024 [41]	Science Monitor: International Medical Journal of Experimental and Clinical Research	Q2
Alrashed et al., 2024 [42]	Advances in Medical Education and Practice	Q2
Alghamdi and Alashban, 2024 [28]	Journal of Radiation Research and Applied Sciences	Q2
Salih, 2024 [43]	Cureus	Q3
Abdelnasser et al., 2025 [32]	Annals of Forest Research	Q3
Alqarni et al. 2024 [29]	Forum for Linguistic Studies	Q3
Gowdar et al., 2024 [44]	Journal of Pharmacy and Bioallied Sciences	Q4
Alshanberi et al., 2024 [45]	Journal of Pharmacy and Bioallied Sciences	Q4
Fadil and Alahmadi, 2024 [46]	Tropical Journal of Pharmaceutical Research	Q4
Alwadaani, 2024 [47]	Majmaah Journal of Health Sciences	Q4
ALruwail et al., 2025 [35]	Forum for Linguistic Studies	Q4

The next step in the quality assessment involved evaluating each study using the JBI’s assessment checklist. The appropriate version of the JBI checklist was applied based on the study design. The average quality scores were 5 ± 0.43 (n=15) for cross-sectional studies and 5.3 ± 1.20 (n=3) for qualitative studies (Tables 5, 6).

**Table 5:** Summary of JBI’s quality assessment of cross-sectional studies JBI: Joanna Briggs Institute

Source	Questions								
	Q1	Q2	Q3	Q4	Q5	Q6	Q7	Q8	Score
Alharbi et al., 2024 [39]	Y	Y	Y	Y	Y	Y	Y	Y	8
Al Shahrani et al., 2024 [30]	Y	Y	Y	Y	N	Y	Y	Y	7
Abdelnasser et al., 2025 [32]	Y	Y	Y	Y	N	Y	Y	Y	7
Almarzouki et al., 2025 [33]	Y	Y	N	Y	N	Y	Y	Y	6
Elhassan et al., 2025 [34]	Y	Y	Y	N	N	Y	Y	Y	6
ALruwail et al., 2025 [35]	Y	Y	Y	Y	N	N	Y	N	5
Alghamdi and Alashban, 2024 [28]	Y	Y	N	Y	Y	N	Y	N	5
Fadil and Alahmadi, 2024 [46]	Y	Y	N	Y	N	N	Y	Y	5
Syed and Alrawi, 2023 [40]	Y	Y	N	Y	N	N	Y	Y	5
Syed et al., 2024 [41]	Y	Y	Y	Y	N	N	N	Y	5
Alshanberi et al., 2024 [45]	Y	Y	Y	N	N	N	Y	N	4
Alwadaani, 2024 [47]	Y	Y	Y	N	N	N	Y	N	4
Alwadaani et al., 2024 [31]	Y	Y	N	Y	N	N	N	N	3
Gowdar et al., 2024 [44]	Y	Y	N	N	N	N	N	N	2
Bin Dahmash et al., 2020 [27]	Y	N	N	N	N	N	Y	N	2

**Table 6:** Summary of JBI’s quality assessment of qualitative studies JBI: Joanna Briggs Institute

Source	Questions										
	Q1	Q2	Q3	Q4	Q5	Q6	Q7	Q8	Q9	Q10	Score
Salih, 2024 [43]	Y	N	Y	N	Y	Y	Y	Y	N	Y	7
Alqarni et al. 2024 [29]	Y	N	N	N	Y	Y	Y	N	Y	Y	6
Alrashed et al., 2024 [42]	N	N	Y	Y	N	N	N	N	N	Y	3

Study Characteristics

Tables 7-8 summarize the characteristics and the key findings of the analyzed studies. Most studies provided complete data on demographic characteristics (e.g., gender, age, and education level). Geographically, 27.8% (5/18) of the studies were conducted in Riyadh [39,27,34,40,41], 16.67% (3/18) in Jeddah [32,33,45], and 11.11% (2/18) in the Eastern Region [47,31]. The remaining studies were conducted either across multiple regions [30,28,42, 46] (22.2%, 4/18) or in one of the following cities: Bishah [29], Aljouf [35], Alkharj [44], or Jazan [43].

**Table 7:** Characteristics of participants in included studies AI: artificial intelligence

Study	Year	City	Study type	Method	Study population	Number of participants	Participants characteristics
Abdelnasser et al. [32]	2025	Jeddah	Cross-sectional study	Online questionnaire	Medical educators, Makkah region	220	126 (57.27%) females and 94 (42.73%) males. 61 (27.7%) lecturers, 53 (24.1%) assistant professors, 53 (24.1%) demonstrators, 33 (15%) associate professors, and 20 (9.1%) professors
Al Shahrani et al. [30]	2024	Multiple regions in Saudi Arabia	Cross-sectional study	Online questionnaire	Medical students	527	300 (52.4%) females and 272 (476%) males. 250 (43.7%) from the western region, 149 (2%) from the central region, 80 (14%) from the eastern region, 56 (9.8%) from the southern region, and 37 (6.5%) from the northern region. The majority of the participants (64.3%) were in clinical years (third to fifth year of the medical program)
Alghamdi and Alashban [28]	2024	Multiple regions in Saudi Arabia	Cross-sectional study	Online questionnaire	Freshly graduated medical students	1,212	Students were recruited from 32 out of 39 Saudi universities
Alharbi et al. [39]	2024	Riyadh	Cross-sectional study	Online questionnaire	Undergraduate healthcare students	354	242 (68.4%) males and 112 (31.6%) females. 39.8% were below 24 years old. 124 (35%) were from the College of Pharmacy, 135 (38.1%) were from the College of Nursing, and 95 (26.8%) were from the College of Emergency Medical Services
Almarzouki et al. [33]	2025	Jeddah	Cross-sectional study	Online questionnaire	Undergraduate medical students	354	226 (63.8%) males and 128 (36.2%) females. Mean age: 21.4 years. 60% were 2nd-year medical students
Alqarni et al. [29]	2024	Bishah	Descriptive study	Online questionnaire	Undergraduate medical students	54	38 (70.4%) males and 16 (29.6%) females. The majority of the participants had limited or moderate experience with AI tools (66.6%), while 22.2% had never used them, and only 11.1% had extensive experience
Alrashed et al. [42]	2024	Multiple regions in Saudi Arabia	Randomized qualitative approach	Semi-structured interviews	Medical students enrolled in the medical education program	13	Age between 20 and 24 years
ALruwail et al. [35]	2025	Aljouf	Cross-sectional study	Online questionnaire	Health science students	384	220 (57.3%) males and 184 (42.7%) females. 174 (45.3%) under 21 and 210 (54.7%) over 21 years
Alshanberi et al. [45]	2024	Jeddah	Cross-sectional study	Online questionnaire	Students and faculty members at a medical college	128	102 (79.9%) females and 26 (20.3%) males. 109 (85.2%) under 25, 10 (7.8%) aged 26-35, and 9 (7%) over 35 years. 116 (90.6%) were students, and 12 (9.4%) were faculty members
Alwadaani [47]	2024	Al-Ahsa	Cross-sectional study	Online questionnaire	Undergraduate medical students and medical practitioners	159	66 (41.5%) were males and 93 (58.5%) were females. Mean age of the participants: 28.9 ± 8.8 years. 102 (64.2%) undergraduates, 19 (11.9%) fellowship, 15 (9.4%) membership, 14 (8.8%) MBBS or equivalent, and 6 (3.8%) postgraduates
Alwadaani et al. [31]	2024	Eastern Region	Cross-sectional study	Online questionnaire	Undergraduate medical students	303	129 (42.57%) females and 174 (57.43%) males. 93.73% of participants were aged between 21-25 years
Bin Dahmash et al. [27]	2020	Riyadh	Cross-sectional study	Online questionnaire	Undergraduate medical students	476	289 (60.5%) males and 187 (39.5%) females
Elhassan et al. [34]	2025	Riyadh	Cross-sectional study	Online questionnaire	Undergraduate medical students	293	95 (32.4%) males and 198 (67.6%) females
Fadil and Alahmadi [46]	2024	Multiple regions in Saudi Arabia	Cross-sectional study	Online questionnaire	Undergraduate medical students	463	175 (37.8%) males and 288 (62.2%) females. 59.6% were between 23-25 years old. 38.4% were between 26-30 years old. 71.3% were from the central region
Gowdar et al. [44]	2024	Alkharj	Cross-sectional study	Online questionnaire	Undergraduate dental students and dental practitioners	100	Dental students
Salih [43]	2024	Jazan	Qualitative case study	Direct interview. Focus group discussions	Faculty members. Undergraduate medical students	45	11 faculty members (7 males and 4 females; mean age: 48.5 years). 34 students (16 males and 18 females; mean age: 22.6 years)
Syed and Alrawi [40]	2023	Riyadh	Cross-sectional study	Online questionnaire	Undergraduate pharmacy students	157	118 (75.2%) males and 39 (24.8%) females. 101 (64.3%) were aged 18-22 years
Syed et al. [41]	2024	Riyadh	Cross-sectional study	Online questionnaire	Researchers and academics	201	140 (69.7%) males and 61 (30.3%) females. 54.2 were 31-35 years old. 43.8% were researchers

**Table 8:** Research objectives and key findings of the analyzed studies AI: artificial intelligence

Author	Year	Main research objectives	Key findings
Abdelnasser et al. [32]	2025	To assess the familiarity level of medical educators with AI and robotics in medical education and healthcare systems. To explore medical educators’ beliefs, perspectives, and expectations regarding the present and future integration of AI and robotics in diverse medical disciplines. To explore the legal liability issues that could arise from the use of AI and robotics in medical education and healthcare systems	Female participants were more familiar with AI, with 63.9% compared to 36.1% of males. They also had a more positive attitude towards integrating AI into medical practice and education. Overall, 70.5% of all participants, regardless of gender, supported adding AI to the medical school curriculum
Al Shahrani et al. [30]	2024	To evaluate the preparedness of medical students in Saudi Arabia regarding AI technologies and their applications. To assess the current state of AI education in Saudi medical colleges and AI use and future perspectives for medical students	Only 14.5% of participants received AI education as part of their curriculum. 34.4% gained AI education through extracurricular activities, mainly self-study. 93.2% had used AI applications before. AI was mostly used for querying medical knowledge, conducting research, and explaining pathologies. 46.5% said AI use had not influenced their specialty choices. Most participants could not define or explain basic AI concepts. Female medical students had higher AI readiness scores. 50% felt confident in assessing information from AI tools
Alghamdi and Alashban [28]	2024	To assess the attitudes and perceptions of freshly graduated Saudi Arabian medical students towards AI utilization in the medical sciences. To evaluate the students’ comprehension of AI principles and the extent to which AI is incorporated into their medical education	83.3% believed AI would play an important role in healthcare. 26% understood the basic computational principles of AI. 56% understood the limitations of AI tools. 69.5% thought all medical students should receive AI training. 8.6% had received AI training
Alharbi et al. [39]	2024	To assess the attitudes, opinions, and perceptions towards ChatGPT among healthcare students	91.2% of participants were familiar with the term "ChatGPT," and 75.1% of them felt comfortable using it in their academic activities. 27.9% found ChatGPT useful for gathering medical information. 87.4% believed ChatGPT had a positive impact on medical education. 60.2% were concerned that ChatGPT may facilitate cheating and plagiarism in academic settings
Almarzouki et al. [33]	2025	To evaluate medical students’ current AI knowledge, exposure, and information sources. To evaluate medical students’ understanding of AI role in the medical field	77.1% of participants reported that they had never taken a course in AI. GPA was not associated with AI use. 78.2% of the participants reported that they knew about AI tools from public media. 18.4% of participants reported that they understood the fundamental basics of AI. 20.1% of the participants reported that their schools offered adequate resources to explore AI applications in medicine
Alqarni et al. [29]	2024	To evaluate Saudi students' perceptions of the effectiveness, reliability, ease of use, preference, and frequency of ChatGPT integration as a tool for English-medium instruction (EMI). To assess Saudi ESP students' perspectives on the use of ChatGPT for learning medical terminology	Participants reported that ChatGPT was effective in helping them understand medical terminology. Participants reported that ChatGPT offered a user-friendly interface that facilitated medical terminology learning
Alrashed et al. [42]	2024	To identify challenges and opportunities associated with the integration of virtual reality (VR), AI, and telemedicine into the Saudi medical curriculum and healthcare system	Participants had mixed opinions on VR, AI, and telemedicine, with some expressing excitement and others showing less interest. Medical students and residents had high expectations for integrating these technologies into education and practice. However, concerns were raised about the preparedness of students, educators, and healthcare professionals to adapt. Many advocated for incorporating VR, AI, and telemedicine modules into the medical curriculum
ALruwail et al. [35]	2025	To assess knowledge, attitude, practice, and related factors toward AI among healthcare science students in northern Saudi Arabia. To examine correlations between knowledge, attitude, and practice	343 (89.3%) of the participants reported that they had not participated in AI courses. Participants showed low knowledge, attitude, and practices about AI applications in medical education. Female participants showed a higher level of knowledge about AI practices
Alshanberi et al. [45]	2024	To analyze the level of AI awareness among medical students	57% learned about AI applications from social media, 7.8% were unaware of AI applications, and the rest gained knowledge from other sources. 77% of participants reported that they were aware of the AI applications in the medical field
Alwadaani [47]	2024	To determine the readiness of medical professionals to implement AI by assessing the knowledge, perceptions, and practice of AI among medical students and doctors	Most of the participants reported that they had not received AI training (86.18%). Participants who received prior AI training showed higher knowledge of AI medical applications than those who had not. Gender had no significant effect on AI knowledge. 56% of postgraduate participants and 85.3% of undergraduate participants reported never applying AI in medical practice. 81.13% of participants reported that AI should be included in the medical curriculum
Alwadaani et al. [31]	2024	To explore undergraduate medical students' views on AI, assess their understanding of AI, and the level of confidence in using basic AI tools in the future	61.72% of participants stated that AI would play an important role in the future. 37.95% of participants stated that they understood the basic computational principles of AI. 59.07% of participants stated that medical students should receive AI training. 15.51% of participants stated that they would be confident in using AI tools in their future career
Bin Dahmash et al. [27]	2020	To assess medical students’ perception of AI and the impact of these perceptions on their choice regarding radiology as a career	50% of participants stated they had a good understanding of AI. However, when assessed, only 25% of those who claimed to have good AI knowledge answered the questions correctly
Elhassan et al. [34]	2025	To examine the familiarity, usage patterns, and attitudes of Alfaisal University medical students toward ChatGPT and other chat-based AI apps in medical education	97.9% of male participants and 90.0% of female participants reported being familiar with AI applications. 46.3% of males and 30.3% of females stated they used ChatGPT and similar AI tools to answer medical questions. 41.1% of males and 31.3% of females reported using AI applications to explain concepts. 58.4% of participants believed AI applications could enhance medical education. 77.1% of participants thought AI applications might encourage academic dishonesty. 70.3% of participants understand that AI-generated content could sometimes be inaccurate. 46.4% of participants considered using AI tools for coursework completion unethical
Fadil and Alahmadi [46]	2024	To evaluate the perceptions, awareness, and opinions of healthcare students towards AI in Saudi Arabia	86.7% of participants stated they had not received any formal AI training. 84.9% expressed concerns about potentially losing their jobs to AI in the future. 40.6% believed AI devalued the medical professions. 70.8% thought AI could help facilitate patient education. 77.5% agreed that AI knowledge and skills should be included in the academic curriculum
Gowdar et al. [44]	2024	To assess the awareness and attitudes of dental students and dental practitioners in Alkharj toward AI	74% of undergraduate dental students reported not being aware of the working principles of AI. 50% of undergraduate dental students were unaware that AI tools could enhance knowledge on a topic. 95% of undergraduate dental students believed AI training should be included in the medical school curriculum
Salih [43]	2024	To explore faculty and students' perspectives on AI, their use of AI applications, and their perspective on its value and impact on medical education	81.8% of faculty members and 85.3% of students stated that they had an AI application installed on their mobile phones/ tablets. Both faculty members and students believed AI would have a positive impact on medical education. However, they expressed concerns that AI tools could provide incorrect information, offer vague references, and pose a threat to academic integrity. Faculty members suggested that course descriptions should be modified to accommodate the use of AI tools and address related concerns
Syed and Alrawi [40]	2023	To determine awareness, perceptions, and opinions toward artificial intelligence among undergraduate pharmacy students	73.9% of participants reported that they knew about AI tools. 69.4% of participants reported that AI tools could help healthcare professionals. 24.8% of participants stated that they could lose their jobs in the future because of AI tools. 10.2% of participants stated that they had received formal training on AI tools. 10.8% of participants stated that AI tools could facilitate patient education
Syed et al. [41]	2024	To assess the awareness and perceptions towards ChatGPT among academicians and researchers in Saudi Arabia	91% of participants reported being familiar with the term ChatGPT. 68.7% of participants expressed a positive attitude towards ChatGPT. 77.1% of participants reported feeling somewhat comfortable using ChatGPT in their health practice, while 15.9% said they were very comfortable. 20.9% of participants had used ChatGPT in their research. 80.1% of participants had asked ChatGPT a question. 85.6% of participants believed that ChatGPT had a positive impact on education

Regarding study population, most of the analyzed studies focused on undergraduate medical students, including those enrolled in colleges of medicine, pharmacy, dentistry, and applied medical sciences [27,28,29,30,31,33,34,35,39,40,42,46]. Two studies exclusively included medical academics and researchers [32,41]. Four studies included both students and medical practitioners or researchers [43,44,45,47].

The earliest study identified in this review was published in 2020 [27]. All studies reported the total number of participants, which ranged from 13 to 1212 individuals. The participants' years of study varied from pre-clinical (years 1, 2, and 3) to clinical years (years 4, 5, and 6) of medical colleges. Their age ranged from 18 to 25 years old. In six studies that included medical academics and professionals, the average age was between 38 and 48 years. The total number of participants of all analyzed studies was 5404 subjects. All analyzed studies provided the gender ratio of participants, with an average of 43.9 ± 4.3% females and 56.07 ± 4.3% males. Research on AI and medical education within Saudi Arabia predominantly employed cross-sectional, questionnaire-based designs (15 out of 18 studies), with a combined sample size of 5,331 subjects. The remaining three studies utilized qualitative descriptive methods [29,42,43], incorporating direct interviews, focus group discussions, and online questionnaires, with a total sample size of 103 subjects.

Medical Students' and Academics' Attitudes Towards AI

In 17 out of 18 analyzed studies, attitudes toward AI tools were measured using various methods: a five-point Likert scale [30,31,33,35,39,28], a three-point Likert scale [32,34,40,41,44], direct interviews and discussion groups [42,43], yes/no questions [45,47], mixed approach using six-point Likert scale and direct interviews [29], or seven-point Likert scale [27]. One study did not measure participants' attitudes towards AI [36].

Cross-sectional studies indicated that both medical students and academics generally have a positive attitude towards integrating AI in medical education, with female participants showing greater enthusiasm compared to their male counterparts. Most studies highlighted that participants believe AI could enhance medical education and improve learning outcomes [27,29,30,32,33,34,35,39,41,42,43,44,45,46,47]. Similarly, qualitative studies involving focus group discussions and direct interviews reported that AI tools would be beneficial to medical education [29,42,43].

Despite these positive findings, some studies raised concerns about the ethical challenges associated with using AI tools in medical education. Participants in these studies expressed concerns about risks to academic integrity, the potential for cheating, and the fear that AI could undermine the value of medical professions [29,32,39,46].

Medical Students' and Academics' Knowledge About AI

Most participants across the analyzed studies reported using AI applications for educational purposes [27,28,29,30,34,35,39,41,42,43,44,45]. However, only a small number had received formal training in AI tools. Their knowledge about AI tools primarily came from extracurricular resources, such as social media and friends. Many studies highlighted gaps in participants’ understanding of basic AI concepts and how AI tools work [27,28,34,42,44].

Additionally, many studies reported that participants had a good understanding of AI tools' limitations, including the potential to generate false information, violate ethical principles, and lack credibility [29,39,43,44,45]. While half of the participants agreed that AI should be integrated into medical education, they also stressed the importance of prioritizing human judgment over AI-generated recommendations. This highlights a key point: students view AI as a valuable tool, but they do not fully trust it [39]. Confidence in using AI is another issue. Although students frequently turn to AI for help, only a minority feel proficient with these tools [28,34,38,40]. This raises an important issue: despite frequent exposure to AI, many students do not fully understand how it works, how reliable it is, or how to critically evaluate its outputs. Many studies suggest that integrating AI education into medical curricula could help address this issue [27,31,31,32,33,34,43,44].

Regarding why medical students use AI tools, most studies reported that they use these tools to look up medical information, answer clinical questions, support research, and better understand complex medical concepts [29,30,35,39,44].

Discussion

This review provides an in-depth analysis of the current literature on the attitudes, knowledge, and practices of medical students and professionals in Saudi Arabia toward integrating AI tools into medical education. Our findings show a generally positive attitude toward AI integration in medical education, yet they also highlight knowledge gaps and concerns about ethical and practical implications.

Interpretation of Key Findings

A key finding from this review is that most of the analyzed studies employed a cross-sectional design, which restricts their ability to identify long-term trends or causal relationships. Nevertheless, the findings consistently indicate that medical students and professionals recognize the advantages of using AI tools in medical education. They stated that AI tools have the potential to enhance the learning experience, improve clinical decision-making, and research capabilities. However, many participants acknowledged the limitations of these tools and emphasized the necessity of human judgment in medical decision-making. Although students often used AI tools for learning, formal training in AI was uncommon. Most students learned about AI tools through social media, peer discussions, or self-study. This points to a significant gap in the medical curriculum that needs to be addressed. Incorporating AI education into the medical curriculum could enable students to gain a better understanding of how these tools function, their benefits, and their limitations. It would also cover crucial topics such as ethics, privacy, data security, and the potential for bias in AI algorithms. This will not only improve the students’ learning experience but also lead to better patient care and treatment outcomes.

Methodological Considerations and Quality of Analyzed Studies

The quality assessment of the studies analyzed revealed several methodological limitations. A recurrent issue was the insufficient control of confounding factors, such as prior exposure to AI technologies, institutional resource disparities, or baseline differences in digital literacy among participants. Many studies relied on self-reported metrics or ad hoc questionnaires lacking psychometric validation. This introduces risks of bias and reduced reliability. Moreover, ambiguities in defining study populations, such as heterogeneous cohorts (e.g., medical students, residents, and practicing clinicians grouped without stratification), compromised the interpretations of outcomes and limited the applicability of findings to specific educational or professional contexts. Additional issues included the poorly designed data collection tools that were either unvalidated or contained multiple questions without clear objectives.

Additionally, there is a lack of mixed-methods research that combines qualitative data with real-world insights from interviews or focus groups. Without this, several key factors, such as how institutions, teaching methods, and faculty attitudes influence AI adoption, will remain unexplored.

The quality of the studies analyzed in this review raises significant concerns, as only 3 out of 18 were published in high-impact (Q1) journals, according to Scopus or Web of Science [34,38,39]. This limited representation in top-tier journals raises questions about the overall rigor and reliability of the research in this field.

AI in education is still an emerging field, with much of the research in its initial phases, which may explain the limited representation in top-tier journals. The research, therefore, might not meet the rigorous standards of high-impact journals, possibly due to insufficient methodologies or sample sizes, raising concerns about the overall reliability and generalizability of the findings.

Additionally, some studies are based on small sample sizes, which reduces their statistical power and limits the ability to generalize findings to larger populations. This also means that some studies may lack the statistical power to detect meaningful effects, even when they are present. This may indicate a need for more rigorous research methodologies and improved study designs. Future research should prioritize hypothesis-driven, intervention-based studies that rigorously evaluate both the cognitive and practical outcomes of AI integration in medical curricula.

Ethical and Practical Challenges

This review highlighted several ethical concerns about using AI in medical education. Participants often expressed worries about maintaining academic integrity, the risk of AI-assisted cheating, and the potential devaluation of medical expertise. These concerns underscore the necessity for regulatory guidelines and ethical frameworks to ensure responsible AI use in medical education. Institutions should establish clear policies on the acceptable use of AI tools in coursework, assessments, and clinical decision-making to address these concerns.

Additionally, there is a clear need to improve students’ confidence in using AI tools. Many participants expressed concerns about their ability to critically assess AI-generated information. This lack of confidence suggests that AI literacy should be integrated into medical training.

Implications for Medical Education

Our findings highlight the importance of integrating AI education into medical curricula. Given that most students acquire AI-related knowledge through informal means, structured AI training programs should be developed to provide foundational knowledge on AI applications, limitations, and ethical considerations.

Additionally, interdisciplinary collaboration between medical educators, AI experts, and policymakers is essential to develop standardized AI curricula that align with global best practices.

Comparison to Global Findings

The findings of this review indicate that medical students generally have a positive attitude toward integrating AI tools into their education. Several survey-based studies have highlighted a diverse range of both positive [48,49,50,51,52,53,54,55,56,57,58] and negative [59,60] attitudes toward AI, reflecting the varied perspectives that healthcare students have about AI technology. Baigi and colleagues (2023) conducted a systematic analysis of 38 studies about the attitudes, knowledge, and skills of medical students towards AI in both high-income countries as well as low- and middle-income countries. They reported that most negative attitudes were found in high-income countries, while positive attitudes dominated in low- and middle-income countries [61]. Nevertheless, an online survey questionnaire, designed for distribution to medical physics professionals and students in both developed and developing countries, reported that 85% of the participants agreed that AI would play a prominent role in the practice of medical physicists. Notably, the majority of those who agreed were from developed countries [53]. These findings suggest that both medical students and educators, whether in developed or developing countries, should receive AI training.

In addition, our findings revealed that the majority of medical students in Saudi Arabia generally had a high level of AI knowledge. However, some medical students reported significant gaps in their understanding of AI technology, which is in line with the findings of Baigi and colleagues (2023). These gaps may be attributed to the lack of structured AI training in their medical programs. To address this, integrating AI-focused courses and practical training into the medical curriculum could substantially improve their understanding of AI in the medical field. There are also ongoing concerns about ethics, data privacy, and the reliability of AI-driven decisions [61].

Future Research Directions

This systematic review identifies several important areas for future research and progress in medical education in Saudi Arabia. A major limitation identified in the existing literature on AI integration in medical education is the lack of studies that involve direct AI interventions. Therefore, future research should investigate the effectiveness of AI applications in improving learning outcomes, enhancing clinical skills, and evaluating their overall impact on medical education. Additionally, there is a need for in-depth studies on the long-term effects of AI, focusing on both the broader scope of medical education and specific learning outcomes. Moreover, there is a growing need for additional research in AI to address the potential risks associated with its integration into the medical education curriculum. As AI technologies and tools become more widespread, issues related to automation bias, over-reliance on technology, data privacy, over- and under-skilling, and the potential for intensifying existing disparities in education should be extensively studied.

Furthermore, AI-based educational studies conducted in Saudi Arabia should adopt well-established guidelines for assessing research quality to ensure robust and reliable data and outcomes. Finally, the integration of AI with interdisciplinary collaboration, merging expertise from various specialties, including healthcare, data science, education, and ethics. This multidisciplinary approach is essential to establish frameworks and evidence-based guidelines for integrating AI into medical curricula.

Limitations

This review has two main limitations. First, many of the included studies were cross-sectional in design and relied on self-reported data, which may introduce response bias and limit the ability to draw causal conclusions. In addition, the diversity in study designs and the tools used to measure outcomes made it difficult to compare findings across studies and prevented a meaningful meta-analysis. Secondly, given the rapid pace at which artificial intelligence is evolving, some of the findings discussed in this review may become outdated soon. Nevertheless, this review offers a useful reference point for future research, particularly in tracking how views on using AI tools in medical education may change over time.

## Conclusions

This systematic review demonstrates that while medical students and professionals in Saudi Arabia generally hold positive views on integrating AI tools into medical education, notable knowledge gaps and ethical concerns still exist. Our findings emphasize the need for more structured AI training within medical programs, clear ethical guidelines for using AI, and further research into the long-term effects of AI on medical training and practice. Addressing these issues is crucial to ensuring that AI becomes a valuable tool in medical education, enhancing learning experiences while upholding academic and professional integrity.

## Appendices

List of excluded studies and reasons for exclusion

Not Specific to Medical Education (n=27)

1. Aldossary et al. (2024). The role of generative AI in education: perceptions of Saudi students. Contemporary Educational Technology, 16, ep536.

2. Wazgar et al. (2022). The use of artificial intelligence and deep learning in medical imaging: a nationwide survey of trainees in Saudi Arabia.‏

3. Faroog et al. (2024). Knowledge and attitude of medical students towards artificial intelligence in ophthalmology in Riyadh, Saudi Arabia: a cross-sectional study. Annals of Medicine and Surgery, 86, 4377-4383.‏

4. Salem et al. (2024). Nursing students’ personality traits and their attitude toward artificial intelligence: a multicenter cross‐sectional study. Journal of Nursing Management, 2024, 6992824.‏

5. Farghaly Abdelaliem et al. (2022). The relationship between nursing students’ smart device addiction and their perception of Artificial Intelligence. Healthcare (11, 110).‏

6. Fazlollahi et al. (2022). Effect of artificial intelligence tutoring vs expert instruction on learning simulated surgical skills among medical students: a randomized clinical trial. JAMA Network Open, 5, e2149008-e2149008.‏

7. Alnasib (2023). Factors affecting faculty members’ readiness to integrate artificial intelligence into their teaching practices: a study from the Saudi higher education context. International Journal of Learning, Teaching and Educational Research, 22, 465-491.‏

8. Mirza et al. (2022). The use of artificial intelligence in medical imaging: a nationwide pilot survey of trainees in Saudi Arabia. Clinics and Practice, 12, 852-866.‏

9. Alsharif et al. (2022). A qualitative study to explore opinions of Saudi Arabian radiologists concerning AI-based applications and their impact on the future of radiology. BJR Open, 4, 20210029.‏

10. Alammari et al. (2024). Beyond boundaries: the role of artificial intelligence in shaping the future careers of medical students in Saudi Arabia. Cureus, 16(9).‏

11. Amin et al. (2024). Utilization of artificial intelligence (AI) in healthcare decision-making processes: perceptions of caregivers in Saudi Arabia. Cureus, 16(8).‏

12. Aljindan et al. (2023). ChatGPT conquers the Saudi medical licensing exam: exploring the accuracy of artificial intelligence in medical knowledge assessment and implications for modern medical education. Cureus, 15(9).‏

13. Hakami et al. (2023). The impact of artificial intelligence on the preference of radiology as a future specialty among medical students at Jazan University, Saudi Arabia: a cross-sectional study. Cureus, 15(7).‏

14. Hazem et al. (2024). Impact of artificial intelligence on medical students’ radiology career choices: a cross-sectional study at King Faisal University, Saudi Arabia. International Journal of Medicine in Developing Countries, 8, 2372-2372.‏

15. Hamd et al. (2023). A closer look at the current knowledge and prospects of artificial intelligence integration in dentistry practice: A cross-sectional study. Heliyon, 9(6).‏

16. Aldowah et al. (2024). Perceptions and knowledge of undergraduate dental students about artificial intelligence in dental schools: a cross-sectional study. The Journal of Contemporary Dental Practice, 25, 148-155.‏

17. Alanizy et al. (2024). Knowledge and attitude of dental students toward the role of artificial intelligence in endodontics in dental clinics at Qassim Region, Saudi Arabia. Journal of International Dental and Medical Research, 17, 1207-1213.‏

18. Meo et al. (2024). Role of artificial intelligence (Google Bard) in morphological, histopathological, and radiological image identifications: Objective Structured Practical Examination (OSPE) type-based performance. Saudi Medical Journal, 45, 531.‏

19. Abou Hashish and Alnajjar (2024). Digital proficiency: assessing knowledge, attitudes, and skills in digital transformation, health literacy, and artificial intelligence among university nursing students. BMC Medical Education, 24, 508.‏

20. Arif (2024). Radiologic technology students’ perceptions on the adoption of artificial intelligence technology in radiology. International Journal of General Medicine, 3129-3136.‏

21. Alsahali (2021). Awareness, views, perceptions, and beliefs of pharmacy interns regarding digital health in Saudi Arabia: a cross-sectional study. JMIR Medical Education, 7, e31149. 

22. Makhlouf et al. (2024). Effectiveness of designing a knowledge-based artificial intelligence chatbot system into a nursing training program: a quasi-experimental design. Nurse Education Today, 137, 106159.‏

23. El-Sayed et al. (2025). The role of artificial intelligence literacy and innovation mindset in shaping nursing students' career and talent self-efficacy. Nurse Education in Practice, 82, 104208.‏

24. Alqahtani et al. (2023). The emergent role of artificial intelligence, natural learning processing, and large language models in higher education and research. Research in Social and Administrative Pharmacy, 19, 1236-1242.‏

25. Yaseen et al. (2025). Assessing nurses’ knowledge regarding the application of artificial intelligence among nursing practice. Nursing Research and Practice, 2025, 9371969.‏

26. Aljefiri et al. (2024). The perception and attitude of the use of robotics among medical students, rehabilitation students, including OT and PT students and specialists, and healthcare faculty members. Journal of Pharmacy and Bioallied Sciences, 16, S3242-S3244.‏

27. Alamer (2023). Medical students’ perspectives on artificial intelligence in radiology: the current understanding and impact on radiology as a future specialty choice. Current Medical Imaging, 19, 921-930.‏

Review Articles (n=10)

1. Alotaibi and Alshehri. Prospects and obstacles in using artificial intelligence in Saudi Arabia higher education institutions. ThePotential of AI-Based LearningOutcomes. Sustainability 2023, 15, 10723.

2. Mir et al. (2023). Application of artificial intelligence in medical education: current scenario and future perspectives. Journal of Advances in Medical Education & Professionalism, 11, 133.‏

3. Sairete et al. (2021). Artificial intelligence: towards digital transformation of life, work, and education. Procedia Computer Science, 194, 1-8.‏

4. Almansour and Alfhaid. Generative artificial intelligence and the personalization of health professional education: a narrative review. Medicine (Baltimore). 2024; 103, e38955.

5. Alqutaibi et al. (2024). Current applications and future perspective of virtual reality in dental education and practice in Saudi Arabia: a scoping review. The Saudi Dental Journal.‏

6. Aldakhil et al. (2024). Awareness and approaches regarding artificial intelligence in dentistry: a scoping review. Cureus, 16(1).‏

7. Iqbal et al. (2021). Review article: impact of artificial intelligence in medical education. MedEdPublish, 10(1).‏

8. Abualrahi et al. (2024). Paradigm shift: a systematic review of integrating artificial intelligence in nursing education. American Journal of Nursing Research, 12, 50-56.

9. Basnawi and Koshak. (2024). Application of artificial intelligence in advanced training and education of emergency medicine doctors: a narrative review. Emergency Care and Medicine, 1, 247-259.‏

10. Feigerlova et al. (2025). A systematic review of the impact of artificial intelligence on educational outcomes in health professions education. BMC Medical Education, 25, 129.‏

Not Related to AI (n=1)

1. Alghamdi et al. (2024). Factors affecting Saudi medical students’ engagement during synchronous and asynchronous eLearning and their impacts on the students’ academic achievement: a national survey. BMC Medical Education, 24, 358.‏

Not Specific to Saudi Arabia (n=8)

1. Allam et al. (2024). Knowledge, attitude, and perception of Arab medical students towards artificial intelligence in medicine and radiology: a multi-national cross-sectional study. European Radiology, 34, 1-14.‏

2. Sallam et al. (2024). Anxiety among medical students regarding generative artificial intelligence models: a pilot descriptive study. International Medical Education, 3, 406-425.‏

3. Zawiah et al. (2023). ChatGPT and clinical training: perception, concerns, and practice of Pharm-D students. Journal of Multidisciplinary Healthcare, 4099-4110.‏

4. AlZaabi et al. (2023). Are physicians and medical students ready for artificial intelligence applications in healthcare?. Digital Health, 9, 20552076231152167.‏

5. Ahmad et al. (2023). Student perspectives on the integration of artificial intelligence into healthcare services. Digital Health, 9, 20552076231174095.

6. Jamal et al. (2023). Integrating ChatGPT in medical education: adapting curricula to cultivate competent physicians for the AI era. Cureus, 15(8).‏

7. Al-Zubaidi et al. (2024). Exploring faculty preparedness for artificial intelligence-driven dental education: a multicentre study. Cureus, 16(7), e64377.

8. Buabbas et al. (2023 ). Investigating students’ perceptions towards artificial intelligence in medical education. Healthcare (11, 1298).

Preprint (n=1)

1. Abouammoh et al. (2023). Exploring perceptions and experiences of ChatGPT in medical education: a qualitative study among medical college faculty and students in Saudi Arabia. MedRxiv, 2023-07.‏
